# Head and neck cancer patients present late cancer cachexia two years after curative chemoradiotherapy

**DOI:** 10.1016/j.clinsp.2025.100851

**Published:** 2025-12-21

**Authors:** Willian das Neves, Thomás Giollo Rivelli, Eduardo Furquim Simão, Marco Aurélio Vamondes Kulcsar, Gilberto de Castro Junior

**Affiliations:** Instituto do Câncer do Estado de São Paulo (ICESP), Hospital das Clínicas, Faculdade de Medicina, Universidade de São Paulo (HCFMUSP), São Paulo, SP, Brazil

**Keywords:** Head neck neoplasms, Sarcopenia, Cachexia, Atrophy, Muscle wasting, Late cachexia

## Abstract

**Introduction:**

Over 90% of Head and Neck Cancers are Squamous Cell Carcinoma (HNSCC). HNSCC patients with no evidence of disease after treatment completion usually present a high frequency of late treatment-related toxicities. Here, we aimed to evaluate the prevalence of cachexia among head and neck squamous cell carcinoma patients treated with cisplatin-based chemoradiation with curative intent and presenting no evidence of disease. We hypothesize that even patients post long-term follow-up and curative-intention treatment present a high frequency of cancer cachexia.

**Materials and methods:**

This cross-sectional observational study included 120 patients with HNSCC who had previously received definitive or adjuvant chemoradiation. Eligible patients were in regular follow-up for at least 2-years, with no evidence of disease. We assessed clinical characteristics, body composition, muscle strength, nutritional status, and blood tests. The primary outcome was the prevalence of cachexia, and the secondary outcome was an association between cachexia and self-reported dysphagia.

**Results:**

Using two different diagnostic criteria (Fearon and Evans criteria), we found that 20.7% and 8.6% of patients presented cancer cachexia after more than two years of follow-up after curative chemoradiation treatment. As expected, cachectic patients presented lower mid-arm muscle circumference, with dysphagia in 73%. In addition, in cachectic patients diagnosed according to Evans's cancer cachexia criteria, we found lower muscle strength levels. There is no association between dysphagia and cancer cachexia, regardless of the diagnostic criteria.

**Conclusion:**

In long-term follow-up, head and neck squamous cell carcinoma patients with no evidence of disease frequently present with cachexia after curative chemoradiation.

## Highlights

• Two diagnostic criteria diagnose cancer cachexia in HNSCC patients.
• Disease-free HNSCC patients present cancer cachexia after long-term follow-up.
• Cachexia is not associated with dysphagia.

## Introduction

More than 90 % of Head and Neck Cancers are Squamous Cell Carcinoma (HNSCC), and approximately 2 % of all cancer-related deaths occur in HNSCC patients. Exposure to tobacco, alcohol, and human papillomavirus infection are the most related causes. More than 60 % of all HNSCC patients are diagnosed with locally advanced disease^1^^,^^2^, and the typical treatment in this scenario is a multimodal treatment that can include surgery followed by adjuvant radiation therapy. Radiation therapy can be delivered as a single modality or combined with cisplatin. In the curative plan, the standard treatment is cisplatin-based concurrent chemoradiotherapy.^1^^,^^2^

The 5-year overall survival rate has been increasing over recent decades, reaching approximately 60 %^3^, ^4^, ^5^, ^6^ due to advances in diagnosis and treatment, improved patient selection, and a multidisciplinary approach. However, HNSCC patients with no evidence of disease after treatment completion usually present a high frequency of late treatment-related toxicities (e.g., dysphagia, xerostomia, hypothyroidism, ototoxicity, osteoradionecrosis, and dental problems, among others)^7^, ^8^, ^9^, negatively impacting survival and quality of life.^10^, ^11^, ^12^

Dysphagia is one of the most common posttreatment symptoms in HNSCC patients (25 %‒67 %)^10^, ^11^, ^12^ and is linked to cancer cachexia. Cancer Cachexia (CC) is a syndrome that is not reversed by food support and is characterized by muscle wasting.^13^ Cachexia pathophysiology is not entirely understood, and cachectic patients show increased systemic inflammation and general metabolic dysfunction, culminating in muscular atrophy, loss of muscular function, and fatigue.^14^^,^^15^

There are several diagnostic criteria for cancer cachexia, most of which are based on body weight loss.^13^^,^^16^ For example, Fearon et al.^13^ defined cachexia based on the presence of weight loss > 5 % over the past six months, Body Mass Index (BMI) < 20 kg/m^2^, and any degree of weight loss > 2 % over the past six months, or sarcopenia. Following these criteria, Jager-Wittenaar et al.^17^ detected a 42 % prevalence of cachexia in newly diagnosed HNSCC patients, which negatively impacted the Quality of Life (QoL), cancer morbidity, and treatment completion, and it was also related to a lower overall survival rate.^17^

Evans diagnostic criteria^16^ defined cachexia based on the presence of a 5 % weight loss in 12-months or less or BMI < 20 kg/m^2^ plus three out of the following five criteria: decreased muscle strength, fatigue, anorexia, low fat-free mass index, or abnormal biochemistry (increased C-reactive protein > 5.0 mg/L; low hemoglobin < 12 g/dL, and low serum albumin < 3.2 g/dL).^16^ In a retrospective study, 361 consecutive patients with HNSCC were evaluated according to the Evans criteria before, immediately after, and at 6- and 12-month posttreatment. The prevalence of cachexia was 6.1 %, 41 %, 18.4 %, and 18.7 %, respectively, and the presence of cachexia in this population was associated with higher chances of cancer recurrence and death.^18^

Although there is some evidence of the prevalence of cancer cachexia after HNSCC treatment completion, there is no data evaluating patients after two years of curative intent treatment. Here, we primarily aimed to assess the cachexia prevalence in HNSCC patients using the two diagnostic criteria after chemoradiotherapy in the curative setting. As a secondary objective, we evaluated the association between cancer cachexia and dysphagia. We hypothesize that even patients post long-term follow-up and curative-intention treatment present a high frequency of cancer cachexia.

## Materials and methods

The Human Research Ethics Committee approved this cross-sectional observational study at the University of São Paulo (CEP-FMUSP; 1.339.832). The recruitment was performed at the Instituto do Cancer do Estado de São Paulo (ICESP; São Paulo, Brazil) between December 2014 and November 2017. All patients met the following inclusion criteria: 1) More than 18y.o.; 2) Histological diagnosis of squamous cell HNSCC, primarily located in the nasopharynx, oropharynx, hypopharynx, larynx, or oral cavity; 3) Received definitive or adjuvant cisplatin-based chemoradiotherapy; 4) Been followed up for at least two years after the end of the treatment; 5) Considered disease-free at the study entry based on clinical, radiological, and endoscopic regular studies; 6) Received regular clinical assessment and monitoring of nutritional support before and during cancer treatment and their follow-up; and 7) Able to read, understand and sign the consent form. The exclusion criteria included 1) Synchronous second tumors and 2) Other primary neoplasms. The authors recruited a total of 120 HNSCC survivors and obtained written informed consent from all patients. Potential sources of bias include survival bias, since only long-term survivors under regular follow-up were included, and recall bias for self-reported symptoms (fatigue, anorexia). Consecutive recruitment and standardized tools were used to mitigate these risks.

Clinical characteristics, body composition, arm circumference, and hand grip strength were evaluated during the inclusion day. Albumin, hemoglobin, and C-Reactive Protein (CRP) were obtained through medical records. Nutritional status was obtained through the Patient-Generated Subjective Global Assessment (PG-SGA) Short Form. No patients were considered malnourished.

### Body composition

Body weight (kg) was measured using electronic scales, and standing height (cm) was measured using a stadiometer. Mid-arm muscle circumference was obtained by measuring the left upper arm, measured at the midpoint between the shoulder and elbow tips.

### Grip strength

Grip strength was measured using a hand dynamometer (Model 5030L1, Indiana, USA). The examiner first demonstrated the procedure and then gave the dynamometer to the subject. After that, the participant was positioned appropriately. The test was performed sitting with the elbow in a 90° flexion. Participants were instructed to exert maximal force. Three attempts with each arm were made at a 90-second interval, and the results were recorded. The maximum force considered was the highest value obtained (kgf).

### Nutritional status

Each patient completed the Patient-Generated Subjective Global Assessment (PG-SGA) Short Form. This questionnaire provides information about weight, food intake, symptoms, functional status, disease state, metabolic stress, and physical nutritional examination. All variables were summed, and the data are presented in a grouped manner. Moreover, the patients were categorized into three groups: A (well-nourished), B (moderately malnourished), and C (severely malnourished).

### Cachexia diagnoses

Patients were classified as cachectic using the following two diagnostic criteria: 1) Fearon et al. (2011): weight loss > 5 % over the past six months (in the absence of simple starvation); BMI < 20 kg/m^2^ and any degree of weight loss > 2 %; or appendicular skeletal muscle index consistent with sarcopenia (mid-arm muscle area) and any degree of weight loss > 2 %^13^. 2) Evans et al. (2008): weight loss of at least 5 % in 12-months or less, or BMI < 20 kg/m^2^, plus three of the following five criteria: decreased muscle strength (defined as low level of strength according to the age)^19^; fatigue (as referred by patients); anorexia (as referred by patients); low fat-free mass index according mid-arm muscle circumference; or abnormal biochemistry (increased C-reactive protein > 5.0 mg/L, low hemoglobin < 12 g/dL, and low serum albumin < 3.2 g/dL).^16^

### Statistical analysis

Analyses were performed in GraphPad Prism, Version 7.0 (GraphPad Software, La Jolla, USA) and IBM SPSS Statistics for Windows, Version 20.0 (IBM Corp, Armonk, USA). All patients were included in descriptive statistics. Patient characteristics are depicted and presented as the mean ± Standard Deviation (SD), range, or frequency and percentage. To compare cachectic and noncachectic patients, the values of each variable were first tested for normal Gaussian distribution using the Shapiro-Wilk normality test and, when appropriate, compared by unpaired two-tailed *t*-test or Mann-Whitney test. Fisher's exact test was used to compare frequencies. Patients with missing data for cachexia were not included in group comparisons. The results were considered statistically significant at *p* < 0.05. No formal sample size calculation was performed. The study included all eligible patients seen during the recruitment period, reflecting real-world clinical practice. This study was reported in accordance with the STROBE guidelines for cross-sectional studies.

## Results

A total of 1940 patients with head and neck squamous cell carcinoma were screened during the study period. After applying the eligibility criteria, 161 were eligible, and 41 refused to participate. Consequently, 120 patients were consecutively included in the analysis. Because the inclusion and exclusion process was straightforward and fully described, we considered that a flow diagram would not add further clarity. The characteristics of the included patients are shown in Table 1. The mean age was 59y.o. (±9.8), and there was a predominance of males (73.6 %). The most common primary site was the oropharynx (41.6 %), followed by the larynx (24.2 %) and oral cavity (19.2 %). Most patients presented with locally advanced disease (T3‒T4, 72.5 %; *N*+, 71.6 %). The mean body weight was 63 kg (±11.8). According to Fearon and Evans's criteria, the overall frequency of cachexia was 20.7 % and 8.6 %, respectively. Although most of the patients complained of some degree of dysphagia (73.3 %), only 3.3 % depended on a feeding tube. The median follow-up from the end of the cancer treatment until the study entry was 42.5 months (24‒125 months).

**Table 1:** Clinical characteristics.

Characteristic	N	%
Total	120	100
Age, mean (SD) (years)	58.4 (10.0)	
Time post treatment completion, mean (range) (months)	42.5 (24‒125)	
Body weight, mean (SD), (kg)	63.3 (13.1)	
Sex		
Male	89	73.6
Female	32	26.4
Primary cancer site		
Oropharynx	50	41.6
Larynx	29	24.2
Oral cavity	23	19.2
Hypopharynx	9	7.5
Nasopharyngea	9	7.5
T staging		
T1	9	7.5
T2	20	16.7
T3	28	23.3
T4	59	49.2
Unknown	4	3.3
N staging		
N0	33	27.5
N1	25	20.8
N2	49	40.8
N3	12	10
Unknown	1	0.8
Feeding tube dependence	4	3.3
Dysphagia	88	73.3
Presence of cachexia		
According to Fearon (2011)^a^	23	20.7
According to Evans (2008)^b^	10	8.6

a 111-patients evaluated for cachexia

b 115-patients evaluated for cachexia.

Table 2 compares non-cachectic and cachectic patients diagnosed according to the Fearon criteria to understand the differences among these populations. Overall, there were no significant differences between cachectic and non-cachectic patients regarding age, muscle strength, or blood tests. However, cachectic patients presented a lower mean mid-arm muscle circumference (24.0 vs. 27.1 cm; *p* < 0.001). In addition, the present study’s data show a statistically significant (*p* < 0.01) higher percentage of moderately malnourished cachectic patients (60.9 %) than non-cachectic patients (10.3 %). There are no cases of severe malnourishment in the studied population, but most cachectic patients were moderately malnourished (64 %). Furthermore, cachectic patients presented a lower mid-arm muscle circumference without decreased muscle strength and no biochemical differences.

**Table 2:** Comparison between cachectic and non-cachectic patients according Fearon (2011).

	Cachectic (*n* = 23)	Non-cachectic (*n* = 88)	p-value
**Age, mean (SD) (years)**	61.5 (9.0)	57.5 (10.1)	0.08
**Muscle strength (kgf)**	26.4 (8.2)	28.0 (8.8)	0.47
**Mid-arm muscle circumference (cm)**	24.0 (2.6)	27.1 (2.9)	< 0.01
**Blood analysis**			
**Albumin, mean (SD) (g/dL)**	4.4 (0.4)	4.6 (0.6)	0.35
**Hemoglobin, mean (SD) (g/dL)**	13.3 (1.4)	13.6 (1.6)	0.39
**C-reactive protein, mean (SD) (mg/L)**	8.5 (10)	7.2 (12.8)	0.65
**Nutritional status**			
Well nourished	9	79	
Moderately malnourished	14	9	< 0.01^a^
Severely malnourished	0	0	

a Fisher exact test.

Cachectic patients evaluated according to Evans criteria showed lower muscle strength and mid-arm muscle circumference than non-cachectic patients (20 kgf and 24.5 cm vs. 28.2 kgf and 26.6 cm, respectively). There were no differences regarding albumin, hemoglobin, and CRP levels. 80 % of the cachectic patients presented a moderate malnourished status compared to 13 % of non-cachectic patients (Table 3). In other words, cachectic patients evaluated with the Evans criteria presented a decrease in muscle mass and muscle function that was not associated with biochemical differences.

**Table 3:** Comparison between cachectic and non-cachectic patients according to Evans (2008).

	Cachectic (*n* = 10)	Non-cachectic (*n* = 105)	p-value
**Age, mean (SD)**	58.7 (6.6)	58.6 (10.0)	0.98
**Muscle strength (kgf)**	20.0 (4.0)	28.2 (8.6)	0.01
**Mid-arm muscle circumference (cm)**	24.5 (2.5)	26.6 (3.0)	0.03
**Blood analysis**			
Albumin, mean (SD) (g/dL)	4.3 (0.3)	4.5 (0.5)	0.22
Hemoglobin, mean (SD) (g/dL)	13.0 (1.3)	13.6 (1.5)	0.29
C-reactive protein, mean (SD) (mg/L)	6.3 (4.3)	7.3 (12.5)	0.78
**Nutritional status**			
Well nourished	2	96	
Moderately malnourished	8	14	< 0.01^a^
Severely malnourished	0	0	

a Fisher exact test.

Table 4 shows the frequency of dysphagia in the studied population according to each cachexia diagnostic criterion. All cachectic patients diagnosed according to Evans's criteria and 74 % of patients characterized as cachectic according to Fearon's criteria manifested dysphagia. However, there was no significant difference associated with the diagnosis of cachexia and the presence of dysphagia, regardless of the cancer cachexia diagnostic criterion. Besides that, there is no association between cancer cachexia and age (< 60, ≥ 60), sex, primary tumor site, and tumor stage (data not shown).

**Table 4:** Cross tabulation to evaluate association between cachexia and dysphagia.

		Cachexia			
Fearon Criteria		Yes	No	Total	p-value
Dysphagia	No	6	23	29	0.96^a^
	Yes	17	65	82	
**Evans Criteria**					
Dysphagia	No	0	30	105	0.06^a^
	Yes	10	75	10	

a Fisher exact test.

## Discussion

Cancer cachexia is the most common cancer-related syndrome. Cachectic patients exhibit poor performance, lower treatment responses rate, and a dismal prognosis.^13^^,^^20^ Indeed, cancer cachexia is present in different types of cancer and different stages and is responsible for approximately 20 % of all cancer deaths.^13^^,^^17^^,^^21^, ^22^, ^23^, ^24^ In this study, after a long-term clinical follow-up, the we detected the prevalence of cachexia according to two standardized diagnostic criteria in disease-free patients, post-cisplatin-based chemoradiotherapy with curative intent. In addition, we demonstrated that cachectic patients presented lower muscle mass and strength, and 73 % of cachectic patients diagnosed according to Fearon’s criteria manifested dysphagia. Furthermore, cachexia, when diagnosed according to both diagnostic criteria, is not associated with dysphagia.

In newly diagnosed HNSCC patients, the cachexia frequency is at least 42 % when diagnosed using the Fearon criteria.^17^ and 6.1 % when diagnosed using the Evans criteria^18^ In the present cohort, the percentages of cachexia were 20.7 % and 8.6 %, according to the Fearon and Evans criteria, respectively. It is essential to highlight that involuntary weight loss in cachexia syndrome is associated with metabolic disturbances related to tumor presence^15^^,^^25^, but the present study comprises only disease-free patients. Kwon and coworkers^18^ showed that cachexia in HNSCC patients increases by 41 % immediately after the end of the treatment compared with pretreatment values (6.1 %). Nevertheless, according to the authors, the cachexia frequency decreases to 18 % after 6- to 12-months post-treatment, approximately twice the frequency we observed two years after treatment completion using Evans criteria.

Regarding dysphagia, we found a high percentage in the cohort. Dysphagia correlates with body and muscle mass loss in cancer and non-cancer patients^26^^,^^27^, which could explain at least part of the present results. These data support the idea that cachexia in disease-free patients could be a side effect of cancer treatment and, as such, is classified as late toxicity. Indeed, approximately 50 % of all head and neck cancer patients present dysphagia after treatment^28^ but do not present cancer cachexia. Cachexia in disease-free patients could also be attributed to a cisplatin side effect, and recent data showing that cisplatin causes muscle mass reduction support this hypothesis.^29^ In addition, no published clinical data from our group support this hypothesis.

Grossberg et al.^30^ recently showed no correlation between body mass loss and survival in radiotherapy-treated HNSCC patients. However, the authors described pre- and post-radiotherapy muscle mass depletion associated with shorter overall survival. The present data suggest that cachexia in disease-free patients has a muscle component. Nevertheless, we did not perform any survival or cancer recurrence analyses, so further studies are needed to elucidate this question better. Severe weight loss is a substantial indicator of cachexia, but hemoglobin and CRP are essential for evaluating metabolic dysfunction, which can trigger weight loss. It is worth noting that cachectic patients in the present cohort did not show differences in metabolic markers compared to noncachectic patients. However, muscle mass and metabolic markers are predictors of mortality in different types of cancer.^31^, ^32^, ^33^

This study had some limitations. However, these limitations also present opportunities for future research. First, performing a more accurate analysis of muscle area using CT scan images was impossible. Second, the authors did not evaluate the impact of cachexia on disease recurrence, survival, and quality of life. Third, other late toxicities (e.g., xerostomia, hypothyroidism, esophageal toxicity, and others) could contribute to cachexia development in the studied patients, and these alterations could also impact muscle mass, strength, biomarkers, and quality of life. Finally, cancer cachexia could be diverse according to sex, primary tumor site, tumor stage, and treatment drug and dosage; however, due to the relatively small sample size, we could not evaluate cancer cachexia prevalence properly according to those different categories.

Although these results reflect patients treated in a large Brazilian cancer center, they may be generalizable to similar populations in tertiary oncology settings, and extrapolation to other geographic regions or healthcare systems should be done cautiously. Furthermore, we believe that larger studies should be conducted to elucidate muscle wasting in head and neck cancer patients after long-term follow-up, using this type of categorization, which could significantly advance the understanding of this complex condition.

## Conclusion

In conclusion, HNSCC patients treated with chemoradiotherapy after at least two years of treatment completion presented with cachexia, and cachectic patients presented with decreased muscle mass. Cancer cachexia is not associated with dysphagia but is related to worse muscle function. Cachexia and metabolic dysfunction should be better studied to elucidate their pathophysiology and prognostic impact on cancer HNSCC survivors to improve patient outcomes.

## Authors’ contributions

Conceptualization: G.C.J., T.G.R. Methodology and Validation: E.F.S. T.G.R., and M.A.V.K. Investigation and project administration: E.F.S. and T.G.R. Formal analysis, data curation, visualization and writing original draft: W.N. Resources and Supervision: G.C.J. Writing review & editing: all authors.

## Funding

This work was supported by the São Paulo Research Foundation (FAPESP), Brazil [grant numbers 2016/20187-6 and 2014/00172-9]. The funding agency had no role in the design of the study; in the collection, analysis, and interpretation of data; in the writing of the report; or in the decision to submit the article for publication.

**Data availability statement:** The de-identified participant data that support the findings of this study are available from the corresponding author upon reasonable request. Data sharing is subject to institutional approvals and a data use agreement, in accordance with the ethics approval (CEP-FMUSP; protocol 1.339.832) and participant privacy considerations. The data are not publicly available due to privacy and ethical restrictions.

## Conflicts of interest

The authors declare no conflicts of interest.
